# Measuring AI Progress in Drug Discovery: A Reproducible Leaderboard for the Tox21 Challenge

**DOI:** 10.1021/acs.jcim.6c01148

**Published:** 2026-09-16

**Authors:** Antonia Ebner, Christoph Bartmann, Sonja Topf, Günter Klambauer, Johannes Schimunek, Sohvi Luukkonen

**Affiliations:** † ELLIS Unit Linz and LIT AI Lab, Institute for Machine Learning, Johannes Kepler University, 4040 Linz, Austria; ‡ Clinical Research Institute for Medical AI, Johannes Kepler University, 4040 Linz, Austria 1

## Abstract

Deep learning has fundamentally transformed fields such as computer vision and natural language processing since the early 2010s and has strongly influenced biomedical research. For drug discovery specifically, a key inflection point—akin to vision’s “ImageNet moment”—arrived in 2015, when deep neural networks surpassed traditional approaches in the Tox21 Data Challenge. This milestone accelerated the adoption of deep learning methods across the pharmaceutical industry, and today, most major companies have integrated these approaches into their research pipelines. Following the conclusion of the Tox21 Challenge, its data set was incorporated into several established benchmarks, including MoleculeNet and the Open Graph Benchmark. However, during these integrations, the data set was altered, and labels were imputed or manufactured, resulting in a loss of comparability across studies. Consequently, the extent to which bioactivity and toxicity prediction methods have improved over the past decade remains unclear. To this end, we introduce a reproducible leaderboard, hosted on Hugging Face with the original Tox21 Challenge data set, together with a set of baseline and representative methods. The current version of the leaderboard indicates that the original Tox21 winner from 2015—the ensemble-based DeepTox method—and the descriptor-based self-normalizing neural networks introduced in 2017 continue to perform competitively and rank among the top methods for toxicity prediction, raising questions about the extent of progress achieved over the past decade. As part of this work, we provide public access to all baselines and evaluated models for inference via standardized API end points on Hugging Face Spaces.

## Graphical abstract

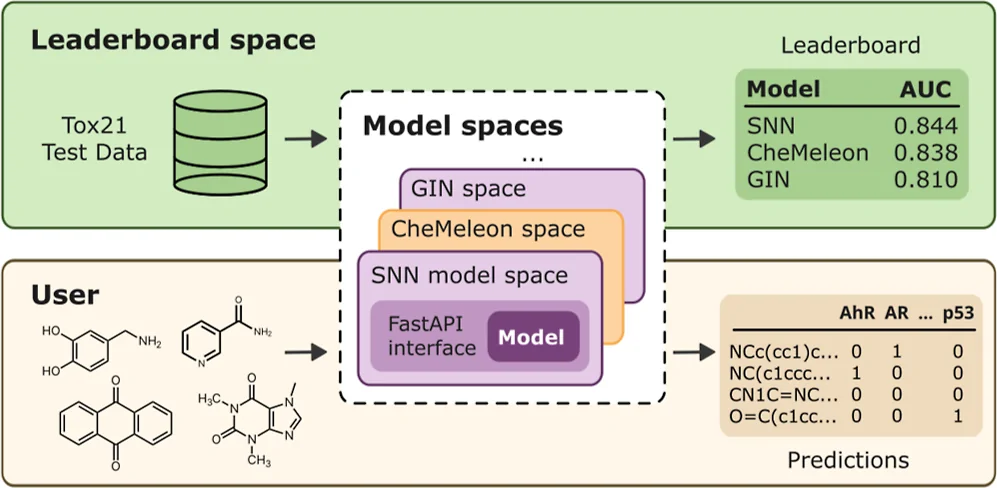

## Introduction

Predicting small-molecule bioactivity, including toxicity, has been a central task in computational drug discovery for decades.^1–3^

During drug development, candidate molecules are experimentally evaluated using in vitro assays that measure biological effects across numerous end points, including target activity, inhibition, and toxicity. However, given the vast pool of candidate molecules,^4–6^ the large number of relevant end points, and the time- and cost-intensive nature of experimental wet-lab testing,^7^ it is infeasible to fully assess all candidates solely through in vitro experimentation. Therefore, computational methods have been developed to prioritize promising compounds and reduce experimental burden.^8–10^ In particular, machine learning and artificial intelligence approaches aim to develop “virtual assays,” in which models predict bioactivity in silico with accuracy comparable to wet-lab experiments.^10,11^ As late-stage clinical failures remain predominantly driven by lack of efficacy and toxicity,^12^ improving predictive performance—especially for toxicity assessment—continues to be a critical objective in drug discovery research.^13^

### “ImageNet Moment” of AI in Drug Discovery

A major milestone for computational methods in drug discovery, particularly for toxicity prediction, was the public release of the Tox21 10K library in 2009. The library initially provided high-quality screening data for approximately 8500 compounds across 70 high-throughput assays and was further expanded and largely completed by 2012. Compared to previous public toxicity screening collections, the Tox21 10K library was orders of magnitude larger and substantially more structurally diverse, providing an unprecedented data resource for developing predictive models.^14^ This data set ultimately enabled the 2015 Tox21 Data Challenge,^15^ in which computational methods were tasked to predict 12 toxicological end points for small molecules.^16^ Somewhat akin to the “ImageNet moment” that accelerated the adoption of deep learning in computer vision in 2012, the challenge marked an important milestone for machine learning in drug discovery, with deep learning emerging as the winning approach. The top-performing method, DeepTox, achieved near-experimental accuracy for predicting mitochondrial membrane disruption, one of the evaluated end points, demonstrating the potential of deep neural networks for toxicity prediction. Although this success did not lead to a widespread replacement of engineered molecular descriptors with learned representations—feature extraction remains a key component of many contemporary workflows and frequently performs comparably to, or better than, learned representations, particularly in low-data settings^17,18^ —it contributed to the growing adoption of deep learning over classical machine learning methods across the field. In subsequent years, deep learning became increasingly prominent in drug discovery research,^19–21^ and many pharmaceutical companies began integrating AI-based approaches into their research and development pipelines.^19,22–24^

### Progress on Toxicity Prediction Remains Unclear

Several benchmark collections have been proposed to standardize evaluation in molecular property prediction. MoleculeNet^25^ provides a unified framework of data sets and evaluation metrics for molecular and quantum-chemical tasks and rapidly became one of the most widely used benchmarks in the field. Other prominent benchmarks for property prediction following MoleculeNet include Therapeutics Data Commons (TDC),^26^ spanning a range of tasks across drug discovery, drug–target interaction, and ADMET prediction;^27^ the Polaris initiative,^28^ providing a benchmarking platform for computational methods in drug discovery; and the Open Graph Benchmark (OGB),^29^ focusing on graph-structured data and enabling large-scale comparisons of graph neural networks. While such benchmark collections have accelerated progress in molecular property prediction by standardizing evaluation across tasks and improving data accessibility, integrating existing data sets into these frameworks has sometimes come at the cost of historical fidelity. One example is Tox21, which was incorporated into several of these benchmarks. During this process, molecules were removed, data set splits were redesigned, and missing labels were replaced with zeros, accompanied by masking schemes (see Benchmark Drift Analysis section). These modifications alter the data set’s statistical properties and prevent direct comparison with results from the original challenge, obscuring the extent of progress achieved over the past decade.

### Centralized Benchmarking Enables Consistent Model Comparisons Across Studies

Recent advances in open infrastructure have made benchmarking increasingly automated and community-driven. Platforms such as the Open LLM Leaderboard^30^ and the LLM Eval Arena^31^ enable model submissions through standardized APIs and assess them within a centralized open-source evaluation framework, ensuring transparency and comparability of results. Similarly, the lm-evaluation-harness^32^ provides a framework for running standardized evaluations of natural language models across a wide range of tasks and data sets. Together, these developments establish reproducible benchmarking workflows that allow models to be evaluated under consistent conditions, reducing discrepancies between studies and enabling continuous, community-driven tracking of methodological progress.

### Re-establishing Faithful Evaluation of Tox21 via an Automated and Reproducible Leaderboard

In this work, we aim to measure the potential progress of toxicity prediction methods by comparing a set of baselines and reference methods on the original Tox21 Data Challenge data set. We aim at high reproducibility and sustainability while allowing the methods maximal freedom in their software environment. To this end, we implement the Tox21 test set as a Hugging Face leaderboard to which methods can be submitted. Our framework interacts directly with Hugging Face Data sets and Spaces to perform automated inference and update leaderboard results. To enter the leaderboard, a method must provide a model card, a reproducible training script, and—critically—expose an API on Hugging Face Spaces that returns predictions for API queries containing small molecules encoded as SMILES strings (see Figure 1). This leaderboard complements existing frameworks such as MoleculeNet, TDC, and OGB by providing a reproducible evaluation hub focused specifically on toxicity prediction. Beyond Tox21, this infrastructure can serve as a blueprint for restoring evaluation consistency across other molecular data sets where benchmark drift has occurred. In the following, we will refer to the original Tox21 Data Challenge data set as Tox21-Challenge and to the MoleculeNet variant as Tox21-MoleculeNet.

**Figure 1:**
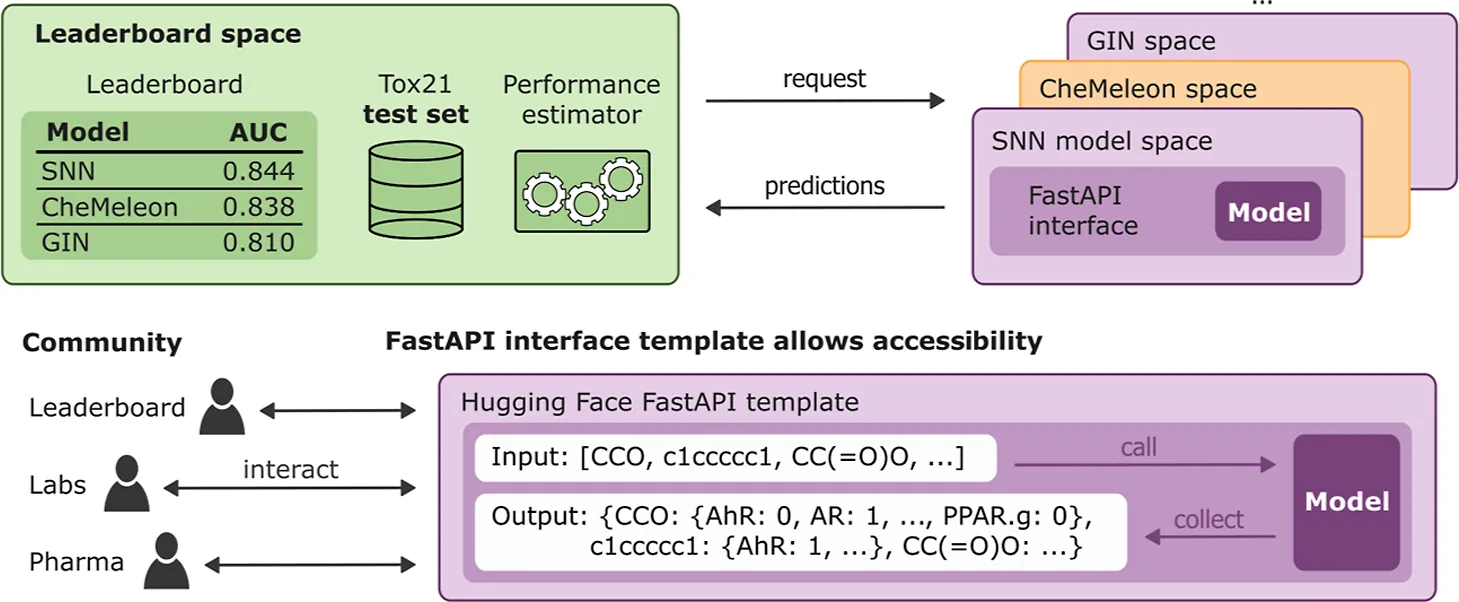
Overview of the Tox21 leaderboard and FastAPI interface linking model spaces with the leaderboard and external users.

## Tox21 Data Set

### Overview

The Tox21 Data Challenge (2014–2015) was a landmark competition for machine learning in drug discovery, as it was one of the first large, public benchmarks for toxicity prediction and catalyzed the use of deep learning in chemoinformatics.^16,33^ It comprised 12 in vitro assays related to human toxicity, spanning across the nuclear receptor (NR) and stress response (SR) pathways. Each assay is framed as a binary classification end point (active vs inactive) for a given target or pathway. The data set was compiled from the U.S. Environmental Protection Agency, National Institutes of Health, and U.S. Food and Drug Administration and contains experimentally validated toxicity measurements.^34^

**Data Set Structure**: The original Tox21-Challenge data set contains 12,060 training samples corresponding to 8043 unique compounds and 647 held-out test samples corresponding to 646 unique compounds. Each sample consists of a compound, represented as a SMILES string, together with annotations for 12 toxicity end points: NR-AR, NR-AR-LBD, NR-AhR, NR-aromatase, NR-ER, NR-ER-LBD, NR-PPAR-γ, SR-ARE, SR-ATAD5, SR-HSE, SR-MMP, and SR-p53. Not all assays were measured for every molecule, and some measurements were inconclusive, resulting in a sparse label matrix with approximately one-third of the entries missing.

**Evaluation**: The official challenge metric was the area under the receiver operating characteristic curve (AUC), computed separately for each end point and averaged across all 12. The organizers performed a train–test split that is challenging due to three factors. First, many test molecules had no structurally similar analogs in the training data, making the setup resemble a cluster-based split. Second, the validation and test assays were conducted in different laboratories than the training assays, introducing interlaboratory measurement variability. Finally, a substantial portion of the test samples was generated during the course of the challenge itself, adding a temporal component to the split that may reflect evolving compound selection and potential sampling bias. Together, these characteristics of the split capture multiple distribution shift sources that are common in molecular property prediction and therefore provide a realistic assessment of model generalization.

### Benchmark Drift Analysis

#### Integration into MoleculeNet and Derived Benchmarks

After the end of the Tox21 Challenge, its data set was integrated into the MoleculeNet benchmark and the DeepChem library^25^ to enable broader model benchmarking. However, several modifications were introduced (see Supporting Information Section S2 for details):

(i) The original 296 validation and 646 test compounds were omitted from the Tox21-MoleculeNet data set and its derivatives.
(ii) Measurements for the same compound were aggregated by taking the maximum label per end point, reducing the data set by 210 samples.
(iii) Three new splitting strategies (random, scaffold-based, and stratified random) replaced the original split and were applied solely to the original training set.
(iv) The data set composition was changed from 8043/296/646 unique compounds (train/validation/test) to 6258/782/783 unique compounds.
(v) The activity distributions in these new test sets differ substantially from the original across all targets, regardless of the splitting strategy.
(vi) In the DeepChem library and TDC benchmark, missing labels were imputed as zeros with an accompanying masking scheme.

Consequently, the resulting Tox21-MoleculeNet data set differs from the original Tox21-Challenge, both in data composition and in evaluation protocol, leading to results that are not directly comparable to those of the original leaderboard. Subsequent frameworks such as TDC^26^ and OGB^29^ adopted the Tox21-MoleculeNet version of Tox21. In the TDC implementation, missing labels were replaced by zeros, and the mask introduced with MoleculeNet^25^ appears to be entirely removed such that unknown assay outcomes are treated as inactive compounds. As a result, many reported Tox21 results are based on altered or corrupted data.

#### Quantifying Benchmark Drift Across Tox21 Data Set Variants

To assess the extent to which training and evaluating using altered or potentially corrupted data set variants affect reported task performance across studies, we trained a single-task Random Forest (RF) classifier^35^ for each Tox21 task separately using either the Tox21-Challenge data set variant or one of the splits of the Tox21-MoleculeNet variant. A single-task setup was chosen because RF classifiers cannot naturally accommodate the missing labels present in the multitask Tox21 data sets. Accordingly, samples with missing labels were excluded on a per-task basis. As RFs are tabular models, molecules were first transformed to a fixed set of molecular features. To reduce bias associated with relying on a single descriptor type, we used a diverse set of descriptors, including ECFP6 fingerprints, MACCS keys, selected RDKit descriptors, and toxicity-related descriptors (see Supporting Information Section S3 for details). To ensure consistency across experiments, no hyperparameter tuning was performed; instead, the same fixed hyperparameter configuration was used for all RF models (see Supporting Information Section S2). Classifiers trained on the Tox21-MoleculeNet variant were evaluated both on their respective test set and on the original Tox21-Challenge test set, while the classifier trained on the Tox21-Challenge variant was evaluated only on its original test set to avoid test set leakage. For each Tox21-MoleculeNet split, we additionally trained a model without applying the intended label masking scheme but evaluated it with the masking scheme applied. We compared the performance of Tox21-MoleculeNet-trained models (i) on their respective test split versus the original test set to quantify discrepancies between reported and comparable cross-study performance; (ii) on the original test set with the performance of the Tox21-Challenge-trained model to assess the effect of data set adaptation; and (iii) with and without the masking scheme to simulate the impact of accidentally using corrupted data. Each experiment was conducted ten times with different random seeds for the RFs while keeping the data set split fixed. Statistical significance was assessed using a two-sided Wilcoxon signed-rank test, with p-values adjusted using the Bonferroni correction to account for multiple testing.

#### Impact of Benchmark Drift

Figure 2 summarizes the experimental results; corresponding p-values are reported in Supporting Information Section S2. Overall, model performance is highly sensitive to the choice of data set variant, splitting strategy, and preprocessing, making results across settings difficult to compare.

**Figure 2:**
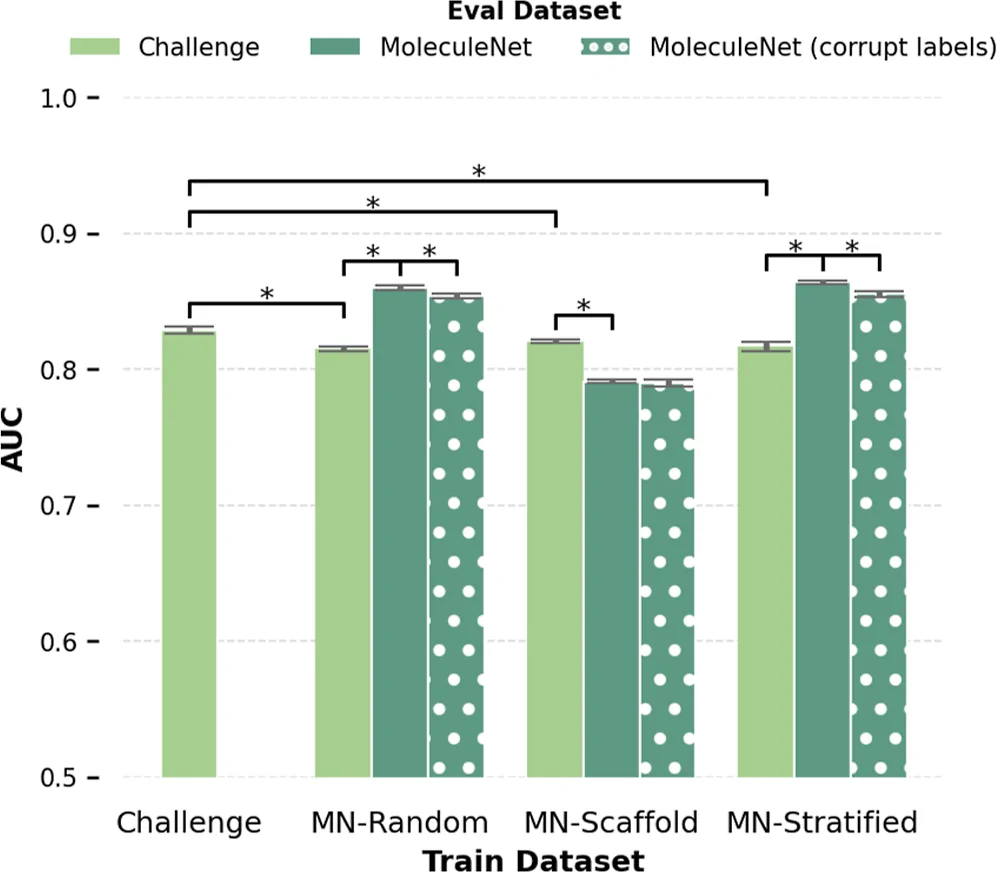
Comparison of test performance of a Random Forest model with fixed configuration trained on the Tox21-Challenge (“Challenge”) data set and the three Tox21-MoleculeNet (“MN”) DeepChem split variants (Random, Scaffold, and Stratified random). Models are evaluated on both their respective test splits and the original Tox21-Challenge test set. Reported values denote the mean test AUC of each model with a standard deviation error bar across ten seeds. Results are shown with correct label masking and without masking, where the latter model is trained without masking, effectively using corrupted labels. An asterisk (*) indicates statistical significance after Bonferroni correction (*p* < 0.05).

As expected, the reported performance of models trained on Tox21-MoleculeNet data set variants differs significantly from their performance on the original Tox21-Challenge test set. Performance on the MoleculeNet test set also varies substantially across splits. These differences largely disappear when the models are evaluated on the original test set.

In contrast, models trained on the original Tox21-Challenge data set consistently achieve higher performance on the original test set than those trained on Tox21-MoleculeNet. This improvement is likely driven by the larger training set and the preservation of the original data distribution, suggesting that informative samples may have been removed or that the learning problem was altered through the use of a maximum-label aggregation strategy during data set adaptation.

These findings indicate that performance metrics obtained on different data set versions or splits are not directly comparable, especially when data set variants and splitting strategies are not explicitly reported. This issue is further amplified by inconsistent benchmark usage, as MoleculeNet recommends random splits^25^ while defaulting to scaffold splits in common implementations.^36^

Finally, omitting the label-masking scheme leads to additional significant performance shifts for two of the three splits, demonstrating how easily results can be distorted when data set-specific preprocessing steps are overlooked. Taken together, these findings highlight the need for consistent and faithful evaluation frameworks to ensure that progress in molecular toxicity prediction can be reliably tracked over time.

## Tox21 Leaderboard

### Implementation

To realign evaluation with the original Tox21 Challenge and enable a standardized and reproducible comparison across models, we implemented an automated leaderboard that executes the original Tox21 evaluation pipeline directly on the original test set: https://huggingface.co/spaces/ml-jku/tox21_leaderboard.

#### Design Principles

The leaderboard design follows three guiding principles: (i) faithful reproduction of the original challenge protocol, (ii) automation and transparency of the evaluation protocol, and (iii) curation and approval to maintain the data integrity. All submissions are evaluated centrally on the fixed, original test set, ensuring that performance values are directly comparable and are unaffected by local preprocessing or metric variations.

#### Infrastructure and Workflow

The leaderboard is hosted as a Hugging Face Space that communicates with user-provided model spaces via FastAPI. To request an evaluation, users submit a model entry containing (i) a link to their public Hugging Face Space exposing a FastAPI end point for inference and (ii) a model card describing architecture, training data, and environment. The request sends the SMILES strings of the 647 locally stored test compounds to the model’s API end point, which returns predictions to the leaderboard backend. The backend computes standardized metrics, and each submission is then verified by the leaderboard administrators (see Supporting Information Section S1.2). Approved results are integrated into a versioned leaderboard data set that is publicly displayed and supports filtering and sorting of models.

#### Evaluation Protocol

Predictions are expected as floating-point values in the range [0,1] for each of the 12 tasks. Performance is measured by the area under the receiver operating characteristic curve (AUC), computed per end point and averaged across tasks, reproducing the original challenge metric. For each model, we report the test AUC together with its 95% confidence interval. The reported confidence intervals quantify the statistical uncertainty of the AUC estimate for a single trained model. For individual tasks, confidence intervals were computed using DeLong’s closed-form method. Confidence intervals for the average AUC across tasks were estimated using bootstrap resampling with 5000 iterations.

To assess whether the observed differences in predictive performance were practically meaningful, we applied the Bayesian Bradley–Terry (BBT) model. The BBT model estimates posterior probabilities of pairwise superiority between methods based on their relative performance across tasks. Each of the 12 Tox21 prediction tasks contributes one observation to the model. We evaluated pairwise comparisons among the five highest-ranked models and compared the top-ranked model against all remaining methods. Following the recommendations of the original authors, a region of practical equivalence (ROPE) of [0.45,0.55] was used to distinguish practically relevant differences from inconclusive or practically equivalent outcomes.^37^ Model fitting and posterior inference were conducted using the bbt-test^38^ package.

#### Tox21 Training Data Set Release

To facilitate reproducible training, we provide the original Tox21-Challenge training data as a Hugging Face data set: https://huggingface.co/datasets/ml-jku/tox21. This data set aligns with the challenge split specification and can be used directly for model development prior to leaderboard submission.

### Models

We re-evaluate a diverse set of representative models spanning descriptor-based, graph-based, and large language approaches. Our lineup includes the original DeepTox method (Tox21 challenge winner),^16^ models trained from scratch, pretrained molecular encoders, pretrained foundation models, and the first external leaderboard submissions. All feature-based models (i.e., RF, XGBoost, SNN, and TabPFN) were trained on the same combination of ECFP6 fingerprints, MACCS keys, RDKit molecular descriptors, and descriptors associated with known toxicity patterns. For details on the model configuration and molecular featurization, see Supporting Information Section S3. Together, these baselines span nearly a decade of methodological evolution in molecular toxicity prediction and lay the foundation for future community contributions and leaderboard benchmarking.

#### Baselines Trained from Scratch

We trained and evaluated a Self-Normalizing Neural Network (SNN)^39^ trained on molecular descriptors, comprising eight fully connected hidden layers with 512 hidden units each; a graph neural network, namely a Graph Isomorphism Network (GIN),^40^ trained directly on molecular graphs (see Supporting Information Section S3.7 for initial node features), consisting of five GINConv layers with a hidden dimension of 128, followed by global mean pooling; and Chemprop,^41^ which combines learned graph features from a directed message passing neural network with molecular descriptors. The Chemprop baseline uses a message-passing depth of 3, a hidden dimension of 400, mean aggregation, and a two-layer feed-forward network with a hidden dimension of 400. We further include classical ensemble methods such as XGBoost^42^ and Random Forests (RF).^35^ Except for DeepTox, all of the models listed above were trained from scratch and underwent extensive hyperparameter optimization.

#### Pretrained Encoders

Following the protocol of Praski et al.,^43^ we additionally evaluated several pretrained molecular encoders. These included the SMILES-based encoders MolBERT,^44^ ChemBERTa-2,^45^ and ChemFM;^46^ the graph-based encoders Molecule Attention Transformer (MAT)^47^ and Relative Molecule Attention Transformer (R-MAT);^48^ and the descriptor-based Contrastive Language-Assay Molecule Pre-Training (CLAMP).^49^ For encoders with multiple publicly available variants, we selected the largest model: ChemFM-3B, MAT-20M, and R-MAT-4M. For ChemBERTa-2, we used the multitask regression (MTR) variant, which predicts multiple molecular descriptors during pretraining, as it has been shown to outperform masked language modeling on downstream molecular property prediction tasks.^43,45^ Among the available MTR models, we selected the variant pretrained on the largest data set (77 M molecules). Details on the encoder architectures are provided in Supporting Information Section S3.10.

For each pretrained encoder, molecule-level embeddings were extracted and used as input features for a separate single-task RF classifier for each of the 12 Tox21 tasks. No task-specific fine-tuning of the pretrained encoder weights was performed. This setup isolates the quality of the learned molecular representations from that of the downstream prediction model. Preliminary experiments using the default RF configuration indicated that only MolBERT and CLAMP achieved competitive validation performance (see Supporting Information Section S4). Consequently, only these two encoders underwent hyperparameter optimization and were subsequently included on the leaderboard.

#### Pretrained Models

We further included three pretrained models: the transformer-based TabPFN (version 2),^50^ which performs inference by conditioning on a labeled support set rather than training on Tox21 data; graph-based GROVER,^51^ which integrates message passing into a transformer-style architecture, pretrained in a self-supervised manner on unlabeled molecular graphs, and fine-tuned on the Tox21 data set using mean graph pooling as the graph readout and a two-layer feed-forward prediction head with a hidden dimension of 13; and a large foundation model baseline, GPT-OSS 120B (high), evaluated in a zero-shot setting on SMILES representations.^52^

#### External Submissions

Since its release, the leaderboard has received multiple model submissions from external developers, which we include in this analysis to further assess current progress. At present, two graph-based approaches have been added: a Directed Message Passing Neural Network (D-MPNN), conceptually equivalent to Chemprop, and CheMeleon,^53^ a foundation model pretrained by predicting molecular descriptors from molecular graphs. In addition, an SNN ensemble method has been submitted, which combines the predictions of ten SNN models using majority voting. The D-MPNN and SNN ensembles were trained from scratch, whereas CheMeleon constitutes an additional pretrained model.

### FastAPI Template for Model Integration and External Access

To facilitate seamless integration of new toxicity prediction models, we provide a minimal FastAPI template that standardizes communication between models and the leaderboard backend. Each model exposes a single/predict end point that accepts a list of SMILES strings and returns a nested JSON dictionary of predictions for the 12 Tox21 targets. The template is lightweight and modular: developers only need to adapt the predict function with model-specific preprocessing and inference code, while the input–output structure remains fixed. This design ensures compatibility with the leaderboard orchestrator and enables uniform access to different model architectures.

Beyond leaderboard evaluation, the FastAPI interface also facilitates external access to toxicity prediction models by other clients, such as pharmaceutical partners or research groups, without requiring them to install or configure the underlying model code. Each provided model space serves as a working example that can be cloned to deploy new models with minimal modification; e.g., see https://huggingface.co/spaces/ml-jku/tox21_gin_classifier. Together, these templates form a reusable interface layer that supports both reproducible benchmarking and real-world integration of molecular prediction models.

### Results and Discussion

Figure 3 summarizes the overall performance of our baseline toxicity prediction models, pretrained encoders, and externally submitted models on the original Tox21 Challenge test set. Detailed results of the BBT test are provided in Supporting Information Section S4.

**Figure 3:**
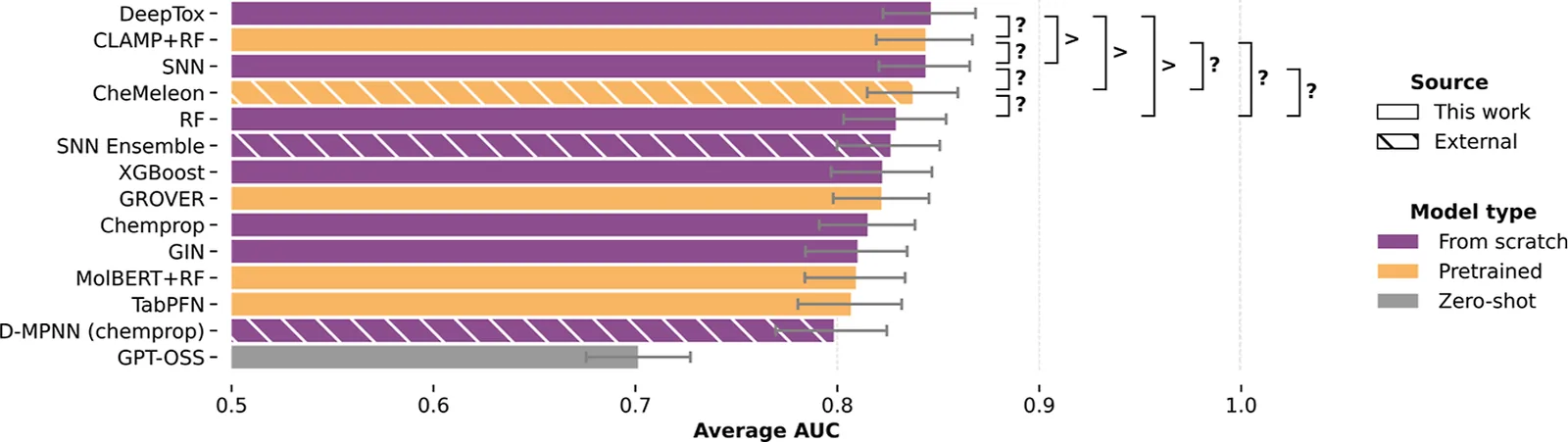
Tox21 model comparison across multiple model types and developers. Reported values denote the average test AUC across the 12 Tox21 end points of each submitted model, together with its 95% confidence interval estimated using bootstrap resampling with 5000 iterations. Brackets indicate the results of the Bayesian Bradley–Terry test (BBT) using the weak interpretation: “>” indicates that the upper model practically outperforms the lower model, whereas ″?″ denotes an inconclusive result.

#### DeepTox Remains the Top-Performing Model Nearly a Decade after the Original Challenge

Among the implemented baselines, pretrained molecular encoders and externally submitted methods, DeepTox achieves the highest overall performance on the current version of the Tox21 leaderboard. Following DeepTox, the CLAMP encoder and the SNN achieve remarkably strong performance, despite their comparatively simple architectures, while DeepTox relies on a large ensemble of neural networks. CheMeleon follows in fourth place and represents the strongest externally submitted as well as pretrained model. RFs operating on a fixed set of molecular descriptors rank fifth and even outperform XGBoost, which is noteworthy since gradient-boosted trees typically dominate in tabular domains, a reversal of the usual trend that has also been reported in a previous Tox21 study.^54^ Among these top five models, the BBT analysis yielded inconclusive results for most pairwise comparisons, indicating that their performances are practically equivalent under the adopted criteria. DeepTox was the only model found to practically outperform multiple competitors, exceeding three of the other four top-ranked methods, while its comparison with the CLAMP encoder remained inconclusive. These results indicate that nearly a decade after winning the Tox21 Challenge, DeepTox remains the highest-ranked model on Tox21 and has not yet been clearly surpassed by more recent approaches. Nevertheless, the current leaderboard represents an initial collection of baseline methods and representative architectures rather than an exhaustive evaluation of the field. We therefore encourage the community to contribute additional models, allowing the leaderboard to serve as a living benchmark for monitoring future advances in computational toxicity prediction.

#### Descriptor-Level Information Provides a Strong Inductive Bias for Toxicity Prediction

A comparison of model performance across input representations and learning paradigms reveals that methods leveraging molecular descriptors—either directly as input features or indirectly through descriptor-predictive self-supervision—consistently outperform purely graph-based approaches. For instance, CheMeleon, a graph neural network trained with descriptor prediction as an auxiliary self-supervised objective, surpasses graph-based methods such as GROVER and GIN, which rely solely on molecular graph representations. Likewise, traditional machine learning models, including Random Forest and XGBoost, which operate on molecular descriptors as input features, also outperform these graph-only approaches. CLAMP represents another high-performing method that incorporates descriptor-level information, ranking second overall.

#### Benefit of Pretraining Remains Inconclusive in the Current Leaderboard

In contrast, the current leaderboard does not reveal a systematic performance advantage of pretrained models over those trained from scratch. While two pretrained models achieved a rank within the top five, four of the eight evaluated pretrained models and encoders using domain-specific pretraining or in-context learning did not achieve an average AUC of 0.8 (see Supporting Information Section S4). Interestingly, both TabPFN and the zero-shot GPT-OSS 120B achieve competitive performance, despite the absence of task-specific training or explicit molecular supervision. In the case of TabPFN, this highlights the potential of pretrained tabular transformers and in-context learning for molecular-property prediction. Likewise, the performance of GPT-OSS 120B suggests that large language models may capture aspects of chemical regularity, even when trained primarily on natural language. As the leaderboard does not yet provide a comprehensive evaluation of the growing landscape of pretrained molecular foundation models, this observation should be interpreted cautiously and may evolve as additional submissions are incorporated. Overall, these findings suggest that descriptor-level information provides a strong inductive bias for toxicity prediction, whereas the benefits of pretraining remain to be inconclusive.

#### Validation–Test Performance Mismatch and Potential Causes

Externally submitted models resembling our baseline models consistently perform worse than their corresponding counterparts despite exhibiting strong validation performance that would suggest better generalization. This discrepancy between the validation and test set performance may be attributable to multiple factors. While the reduced performance of the SNN ensemble may be partly due to its use of the Tox21-MoleculeNet data set variant for training (see Benchmark Drift Analysis section), a major contributing factor may be either insufficient or excessive hyperparameter optimization, with the latter potentially leading to overfitting on the validation set. Although the original validation set follows a similar splitting scheme to the test set with respect to interlaboratory variability, it appears to be a poor predictor of test performance, potentially due to its limited size. This limitation likely motivated the use of multiple cross-validation splits within the training set in DeepTox.^16^ As our baselines used one of these cross-validation splits for hyperparameter tuning, this might lead to better generalization than those obtained by externally submitted models.

## Conclusion and Outlook

The presented leaderboard restores faithful evaluation of the original Tox21-Challenge and enables reproducible comparison of bioactivity models under identical conditions. By combining historical fidelity with automated benchmarking on Hugging Face Spaces, it addresses long-standing inconsistencies in the evaluation of molecular toxicity models. Our findings indicate that it remains unclear how much genuine progress has been achieved over the past decade, as a method proposed in 2014 still performs competitively on the original benchmark. This observation suggests that similar re-evaluations may be warranted for other bioactivity prediction end points such as SIDER^55^ or MUV,^56^ where benchmark drift may likewise obscure scientific progress. Beyond serving as a reliable reference for supervised toxicity prediction, the framework can be readily extended to few-shot and zero-shot evaluation settings, as illustrated by our pretrained baselines. Such extensions will allow systematic assessment of foundation models and in-context learning approaches for molecular prediction,^57–60^ contributing to a larger landscape of bioactivity model evaluation.

## Supplementary Material



## Data Availability

The HuggingFace leaderboard is available at https://huggingface.co/spaces/ml-jku/tox21_leaderboard. The data used to train the submitted models can be accessed at https://huggingface.co/spaces/ml-jku/tox21. The code for the trained baseline models is publicly available in their respective HuggingFace Spaces, collected at https://huggingface.co/collections/ml-jku/tox21-leaderboard-and-model-spaces.

## Notes

The following files are available free of charge. The Supporting Information is available free of charge at https://pubs.acs.org/doi/10.1021/acs.jcim.6c01148.

Specification of leaderboard submissions; analysis of Tox21 data set variants; baseline training and data specification; additional results; and FastAPI template details (PDF)
