# Supplementary material for: Measuring AI Progress in Drug Discovery: A Reproducible Leaderboard for the Tox21 Challenge
Source: J Chem Inf Model. 2026 Sep 16;66(18):11547–55. doi: 10.1021/acs.jcim.6c01148 (PMC13625945; doi:10.1021/acs.jcim.6c01148)
Supplement: Supplementary file 1 [file ci6c01148_si_001.pdf]

Supporting Information - Measuring AI Progress in Drug Discovery: A Reproducible Leaderboard for the Tox21 Challenge

Antonia Ebner[1,2], Christoph Bartmann[1], Sonja Topf[1,2], Günter Klambauer[1,2], Johannes Schimunek[1], and Sohvi Luukkonen*[1]

[1]ELLIS Unit Linz and LIT AI Lab, Institute for Machine Learning, Johannes Kepler University, 4040 Linz, Austria
[2]Clinical Research Institute for Medical AI, Johannes Kepler University, 4040 Linz, Austria

*Email: luukkonen@ml.jku.at

S1 Specification of Leaderboard Submissions

S1.1 User Request to Add a Model

To integrate a new model into the leaderboard, users submit an evaluation request through the leaderboard frontend using a standardized submission template. Each submission must include a model card following the guidelines of Mitchell et al.[1], along with metadata specifying the model's Hugging Face Space URL and corresponding Git commit hash:

Model card

Model details

- Model name:
- Developer:
- Paper URL:
- Model type / architecture:
- Inference:
- Model version:
- Model date:
- Reproducibility: Code for full training is available and enables reproducible retraining of the model from scratch.

Intended use:

Metric:

Training data:

Evaluation data:

Hugging Face Space:

The Hugging Face Space repository must include all code necessary to reproduce the submitted model from scratch.

Submitted models must provide predictions for all 647 test molecules and all twelve Tox21 targets. The leaderboard validates that predictions for every molecule–target pair are present and raises an error otherwise. This ensures a fair comparison between models and prevents potential test score hacking by excluding difficult molecules.

S1.2 Submission Verification Step

The Tox21 leaderboard emphasizes reproducibility. To ensure this, all submissions are manually reviewed before being displayed. Users first submit an evaluation request and provide all required information. The leaderboard automatically retrieves and evaluates the submitted model's predictions, and subsequently marks the obtained performance results as preliminary. After a manual review — verifying (a) completeness of the provided information and (b) reproducibility of model training – the evaluation and its results are marked as approved and subsequently published on the leaderboard.

S2 Analysis of Tox21 Dataset Variants

Table S1 lists the twelve targets included in the Tox21 dataset, together with short descriptions.

Table S1: Tox21 assay targets and descriptions

**Target**	**Description**
NR-AR	Androgen Receptor - involved in male hormone signaling
NR-AR-LBD	Androgen Receptor Ligand Binding Domain - direct binding to androgen receptor
NR-AhR	Aryl Hydrocarbon Receptor - responds to environmental chemicals
NR-Aromatase	Aromatase enzyme - converts androgens to estrogens
NR-ER	Estrogen Receptor - involved in female hormone signaling
NR-ER-LBD	Estrogen Receptor Ligand Binding Domain - direct binding to estrogen receptor
NR-PPAR-gamma	Peroxisome Proliferator-Activated Receptor Gamma - regulates metabolism
SR-ARE	Antioxidant Response Element - responds to oxidative stress
SR-ATAD5	ATAD5 - involved in DNA replication and genome stability
SR-HSE	Heat Shock Response Element - responds to cellular stress
SR-MMP	Mitochondrial Membrane Potential - indicates mitochondrial function
SR-p53	p53 tumor suppressor - activated by DNA damage and stress

Figure S1 summarizes the overlap of unique molecules between the original Tox21-Challenge splits and the full Tox21-MoleculeNet dataset across multiple preprocessing pipelines. The figure shows that the MoleculeNet[2] version only contains molecules present in the original training split, but none unique to the validation or test split. Nevertheless, a subset of molecules in the MoleculeNet dataset cannot be directly mapped to entries in the original dataset under standard preprocessing. This discrepancy is largely resolved after removing stereochemical information, leaving only two unmappable molecules. This suggests that most mismatches arise from differences in stereochemistry rather than molecule grouping, and that stereochemical information may have been modified, removed, or reassigned during MoleculeNet's preprocessing. However, as their full preprocessing pipeline is not publicly available, the precise origin of these discrepancies remains unclear.

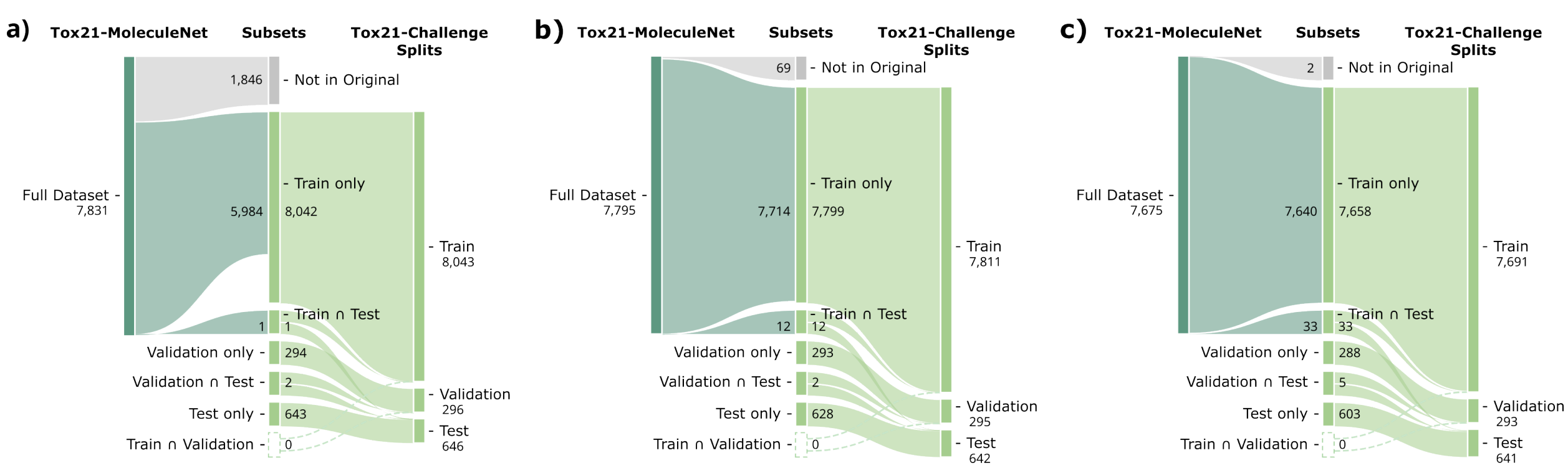

Figure S1: Overlap analysis of Tox21-MoleculeNet and each split of the original Tox21-Challenge dataset across three preprocessing settings: a) no preprocessing of SMILES, (b) canonicalization with salt removal, and (c) canonicalization with salt and stereochemistry removal. Values denote unique SMILES strings. "Subsets" correspond to molecule subsets of the Tox21-Challenge splits. For example, "Train only" includes all molecules present in the training set that do not appear in either the validation or test set, while "Train ∩ Test" contains molecules present in both training and test set.

Table S2 compares the original Tox21 dataset with the preprocessed versions distributed through MoleculeNet and DeepChem[3]. The redistributed data contains fewer samples overall and exhibits substantial differences in train/validation/test splits, percentages of missing values, and active compound ratios compared to the original dataset.

Table S2: Comprehensive comparison of Tox21 dataset splits. "Original" refers to the data as released in the original challenge, whereas "MN" denotes the MoleculeNet version using DeepChem splits (Random, Scaffold, and Stratified random).

Split	Dataset	Total	Unique	Labeled %	Missing %	Active %
Train	Original	11764	8043	69.7	30.3	7.3
	MN-Random	6258	6258	82.8	17.2	7.4
	MN-Scaffold	6258	6258	84.7	15.3	7.1
	MN-Stratified	6258	6258	83.2	16.8	7.5
Validation	Original	296	296	88.1	11.9	8.2
	MN-Random	782	782	83.3	16.7	7.5
	MN-Scaffold	782	782	76.1	23.9	9.7
	MN-Stratified	782	782	81.1	18.9	7.7
Test	Original	647	646	89.8	10.2	7.0
	MN-Random	783	783	83.8	16.2	8.3
	MN-Scaffold	783	783	76.1	23.9	9.3
	MN-Stratified	783	783	82.4	17.6	7.6

To further illustrate these discrepancies, Table S3 provides a detailed breakdown of data availability and class balance for each assay across all dataset variants and splits. The variation in missing value percentages and activity ratios across splits highlights potential issues with data preprocessing and splitting strategies in the redistributed versions.

Random Forest Setup for Benchmark Drift Evaluation. For each dataset split, we trained a separate single-task Random Forest model per Tox21 target using the `scikit-learn` implementation. We employed the same featurization procedure as described in Section S3.1. No hyperparameter tuning was performed. Instead, we used a fixed configuration with `n_estimators=1000` and otherwise default settings. The code used for all experiments is publicly available at https://huggingface.co/spaces/ml-jku/tox21_rf_classifier/blob/main/README.md.

Table S4: Results of the two-sided Wilcoxon signed-rank tests with Bonferroni-adjusted p-values. Setting A and Setting B denote the training and evaluation datasets used for models A and B, respectively. "Original" refers to the data as released in the original challenge, whereas "MN" denotes the MoleculeNet version using DeepChem splits (Random, Scaffold, and Stratified random). P-values are rounded to four decimal places.

Setting A		Setting B		Adjusted p-value
Train Dataset	Test Dataset	Train Dataset	Test Dataset	
Challenge	Challenge	MN-Random	Challenge	0.0039
Challenge	Challenge	MN-Scaffold	Challenge	0.0039
Challenge	Challenge	MN-Stratified	Challenge	0.0039
MN-Random	Challenge	MN-Random	MN-Random	0.0039
MN-Scaffold	Challenge	MN-Scaffold	MN-Scaffold	0.0039
MN-Stratified	Challenge	MN-Stratified	MN-Stratified	0.0039
MN-Random	MN-Random	MN-Random (corrupt labels)	MN-Random	0.0039
MN-Scaffold	MN-Scaffold	MN-Scaffold (corrupt labels)	MN-Scaffold	0.4258
MN-Stratified	MN-Stratified	MN-Stratified (corrupt labels)	MN-Stratified	0.0039

Table S3: Comparison of label availability, missing data, and class balance across train/validation/test splits for all Tox21 assays. Statistics shown for the original dataset and MoleculeNet ("MN") version, employing three DeepChem splitting strategies (Random, Scaffold, Stratified random). Lab.% = labeled samples, Miss.% = missing values, Act.% = active compounds.

Assay	Dataset	Train			Validation			Test		
		Lab.%	Miss.%	Act.%	Lab.%	Miss.%	Act.%	Lab.%	Miss.%	Act.%
NR-AR	Original	79.6	20.4	4.1	98.6	1.4	1.0	90.6	9.4	2.0
	MN-Random	92.6	7.4	4.2	93.5	6.5	3.8	93.7	6.3	4.6
	MN-Scaffold	93.0	7.0	4.3	92.5	7.5	4.3	91.3	8.7	3.8
	MN-Stratified	93.0	7.0	4.2	91.2	8.8	4.3	92.6	7.4	4.3
NR-AR-LBD	Original	73.1	26.9	3.5	85.5	14.5	1.6	90.0	10.0	1.4
	MN-Random	86.1	13.9	3.4	87.1	12.9	4.6	86.7	13.3	3.4
	MN-Scaffold	87.9	12.1	3.5	80.2	19.8	4.0	79.7	20.3	3.0
	MN-Stratified	86.5	13.5	3.5	84.4	15.6	3.6	86.5	13.5	3.5
NR-AhR	Original	69.4	30.6	11.6	91.9	8.1	11.4	94.3	5.7	12.0
	MN-Random	83.6	16.4	11.7	84.3	15.7	11.4	83.5	16.5	12.4
	MN-Scaffold	84.6	15.4	11.1	79.2	20.8	14.1	80.3	19.7	14.6
	MN-Stratified	84.0	16.0	11.7	82.2	17.8	12.0	82.0	18.0	12.0
NR-Aromatase	Original	61.4	38.6	5.0	72.3	27.7	8.4	81.6	18.4	7.4
	MN-Random	74.0	26.0	4.8	75.7	24.3	5.6	75.6	24.4	7.3
	MN-Scaffold	76.4	23.6	4.3	65.1	34.9	8.8	66.8	33.2	9.0
	MN-Stratified	75.0	25.0	5.1	69.4	30.6	5.5	73.7	26.3	5.2
NR-ER	Original	65.4	34.6	12.2	89.5	10.5	10.2	79.8	20.2	9.9
	MN-Random	79.3	20.7	12.4	76.5	23.5	13.2	79.8	20.2	15.2
	MN-Scaffold	81.1	18.9	12.7	70.8	29.2	13.5	70.8	29.2	12.6
	MN-Stratified	79.1	20.9	12.8	77.2	22.8	13.1	80.8	19.2	12.5
NR-ER-LBD	Original	74.4	25.6	5.1	97.0	3.0	3.5	92.7	7.3	3.3
	MN-Random	88.7	11.3	4.9	89.0	11.0	5.5	89.9	10.1	5.7
	MN-Scaffold	90.0	10.0	5.3	84.9	15.1	4.4	83.4	16.6	3.2
	MN-Stratified	89.1	10.9	5.0	87.6	12.4	5.1	87.4	12.6	5.1
NR-PPAR-gamma	Original	69.6	30.4	2.7	90.2	9.8	5.6	93.5	6.5	5.1
	MN-Random	82.2	17.8	2.9	82.7	17.3	2.9	83.1	16.9	3.1
	MN-Scaffold	84.6	15.4	2.5	73.7	26.3	5.6	73.4	26.6	3.8
	MN-Stratified	82.7	17.3	2.9	81.2	18.8	3.0	81.0	19.0	3.0
SR-ARE	Original	60.9	39.1	15.3	79.1	20.9	20.5	85.8	14.2	16.8
	MN-Random	74.5	25.5	16.3	74.6	25.4	14.2	74.1	25.9	17.4
	MN-Scaffold	77.8	22.2	14.7	60.5	39.5	22.4	61.4	38.6	24.5
	MN-Stratified	74.4	25.6	16.2	75.3	24.7	16.0	74.1	25.9	16.2
SR-ATAD5	Original	77.3	22.7	3.7	91.9	8.1	9.2	96.1	3.9	6.1
	MN-Random	90.1	9.9	3.7	91.2	8.8	3.9	91.3	8.7	3.9
	MN-Scaffold	91.5	8.5	3.4	84.9	15.1	5.3	85.8	14.2	4.9
	MN-Stratified	90.7	9.3	3.7	88.0	12.0	3.8	89.4	10.6	3.7
SR-HSE	Original	69.3	30.7	5.3	90.2	9.8	3.7	94.3	5.7	3.6
	MN-Random	82.3	17.7	5.7	84.3	15.7	5.2	82.8	17.2	6.6
	MN-Scaffold	84.9	15.1	5.3	73.4	26.6	7.7	73.1	26.9	8.2
	MN-Stratified	82.5	17.5	5.8	82.6	17.4	5.7	82.9	17.1	5.7
SR-MMP	Original	62.2	37.8	15.6	80.4	19.6	16.0	83.9	16.1	11.0
	MN-Random	73.8	26.2	15.8	73.5	26.5	15.8	77.7	22.3	15.6
	MN-Scaffold	76.3	23.7	14.9	65.0	35.0	21.9	66.4	33.6	18.5
	MN-Stratified	74.8	25.2	15.7	70.5	29.5	16.7	73.2	26.8	16.1
SR-p53	Original	73.4	26.6	6.2	90.9	9.1	10.4	95.2	4.8	6.7
	MN-Random	86.4	13.6	6.0	87.0	13.0	7.1	87.2	12.8	7.2
	MN-Scaffold	87.7	12.3	5.0	82.9	17.1	11.6	80.5	19.5	11.4
	MN-Stratified	86.9	13.1	6.2	83.6	16.4	6.4	85.8	14.2	6.2

S3 Baseline Training and Data Specification

S3.1 Molecule Features

Feature-based bioactivity models are sensitive to the choice of molecular representation[4,5]. To isolate architectural effects, we fixed the input features across all feature-based baselines (RF, XGBoost, SNN, TabPFNv2) and used the same 9,385 molecular features for every model. The final input vector was a concatenation of:

- 8192-bit folded ECFP6 count fingerprints,
- 166 MACCS keys,
- 200 selected RDKit molecular descriptors, and
- 827 descriptors associated with known toxicity patterns.

The selection of RDKit descriptors was fixed across models and is specified in the Hugging Face Space for each feature-based baseline. Before modeling, we optionally applied low-variance and high-correlation feature filtering, quantization of RDKit descriptors, and feature normalization. The corresponding respective variance and correlation thresholds, along with flags enabling quantilization and normalization, were treated as hyperparameters to be optimized for each model. For TabPFN, we only used the 500 highest-variance features after filtering, as TabPFN supports at most 500 features.

S3.2 Self-Normalizing Neural Network

Model card – SNN

Model details

- **Model name:** Self-Normalizing Neural Network Tox21 Baseline
- **Developer:** JKU Linz
- **Paper URL:** https://proceedings.neurips.cc/paper_files/paper/2017/hash/5d44ee6f2c3f71b73125876103c8f6c4-Abstract.html
- **Model type / architecture:**
  - Self-Normalizing Neural Network implemented using `PyTorch`.
  - **Hyperparameters:** https://huggingface.co/spaces/ml-jku/tox21_snn_classifier/blob/main/config/config.json
  - A multitask network is trained for all Tox21 targets.
- **Inference:** Access via FastAPI endpoint. Upon receiving a Tox21 prediction request, the model generates and returns predictions for all Tox21 targets simultaneously.
- **Model version:** v0
- **Model date:** 14.10.2025
- **Reproducibility:** Code for full training is available and enables reproducible retraining of the model from scratch.

Intended use:
This model serves as a baseline benchmark for evaluating and comparing toxicity prediction methods across the 12 pathway assays of the Tox21 dataset. It is not intended for clinical decision-making without experimental validation.

Metric:
Each Tox21 task is evaluated using the area under the receiver operating characteristic curve (AUC). Overall performance is reported as the mean AUC across all individual tasks.

Training data:
Tox21 training and validation sets.
Evaluation data:
Tox21 test set.

Hugging Face Space:
https://huggingface.co/spaces/ml-jku/tox21_snn_classifier/blob/main/README.md

S3.3 Random Forest

Model card – RF

Model details

- **Model name:** Random Forest Tox21 Baseline
- **Developer:** JKU Linz
- **Paper URL:** https://link.springer.com/article/10.1023/A:1010933404324
- **Model type / architecture:**
  - Random Forest implemented using `sklearn.RandomForestClassifier`.
  - **Hyperparameters:** https://huggingface.co/spaces/ml-jku/tox21_rf_classifier/blob/main/config/config.json
  - A separate single-task RF is trained for each Tox21 target.
- **Inference:** Access via FastAPI. Upon a Tox21 prediction request, a target-specific RF model is called separately for each target; outputs are collected across all single-task models and returned.
- **Model version:** v0
- **Model date:** 14.10.2025
- **Reproducibility:** Code for full training is available and enables retraining from scratch.

Intended use:
This model serves as a baseline for evaluating and comparing toxicity prediction methods across the 12 Tox21 pathway assays. It is not intended for clinical decision-making without experimental validation.

Metric:
Each Tox21 task is evaluated using the area under the receiver operating characteristic curve (AUC). Overall performance is reported as the mean AUC across all tasks.

Training data:
Tox21 training and validation sets.
Evaluation data:
Tox21 test set.

Hugging Face Space:
https://huggingface.co/spaces/ml-jku/tox21_rf_classifier/blob/main/README.md

S3.4 XGBoost

Model card – XGBoost

Model details

- **Model name:** XGBoost Tox21 Baseline
- **Developer:** JKU Linz
- **Paper URL:** https://cran.ms.unimelb.edu.au/web/packages/xgboost/vignettes/xgboost.pdf
- **Model type / architecture:**
  - Extreme Gradient Boosting implemented using `xgboost`.
  - **Hyperparameters:** https://huggingface.co/spaces/ml-jku/tox21_xgboost_classifier/blob/main/config/config.json
  - A separate single-task XGBoost classifier is trained for each Tox21 target.

- **Inference:** Access via FastAPI endpoint. Upon receiving a Tox21 prediction request, the model generates and returns predictions for all Tox21 targets simultaneously.
- **Model version:** v0
- **Model date:** 14.10.2025
- **Reproducibility:** Code for full training is available and enables reproducible retraining of the model from scratch.

Intended use:
This model serves as a baseline benchmark for evaluating and comparing toxicity prediction methods across the 12 pathway assays of the Tox21 dataset. It is not intended for clinical decision-making without experimental validation.

Metric:
Each Tox21 task is evaluated using the area under the receiver operating characteristic curve (AUC). Overall performance is reported as the mean AUC across all individual tasks.

Training data:
Tox21 training and validation sets.
Evaluation data:
Tox21 test set.

Hugging Face Space:
https://huggingface.co/spaces/ml-jku/tox21_xgboost_classifier/blob/main/README.md

S3.5 GROVER

Model card – GROVER

Model details

- **Model name:** Grover Tox21 Baseline
- **Developer:** Tencent AI Lab (finetuned by JKU Linz)
- **Paper URL:** https://arxiv.org/pdf/2007.02835
- **Model type / architecture:**
  - Grover implemented using the code accompanying the paper.
  - The pretrained `grover_base` model is used as provided.
  - **Hyperparameters:** https://huggingface.co/spaces/ml-jku/tox21_grover_classifier/blob/main/config/config.json
  - A multitask network is finetuned for all Tox21 targets.
- **Inference:** Access via FastAPI endpoint. Upon receiving a Tox21 prediction request, the model generates and returns predictions for all Tox21 targets simultaneously.
- **Model version:** v0
- **Model date:** 10.12.2025
- **Reproducibility:** Code for full training is available and enables retraining of the model.

Intended use:
This model serves as a baseline benchmark for evaluating and comparing toxicity prediction methods across the 12 pathway assays of the Tox21 dataset. It is not intended for clinical decision-making without experimental validation. **Metric:**

Each Tox21 task is evaluated using the area under the receiver operating characteristic curve (AUC). Overall performance is reported as the mean AUC across all individual tasks.

Training data:
Tox21 training and validation sets.
Evaluation data:
Tox21 test set.

Hugging Face Space:
https://huggingface.co/spaces/ml-jku/tox21_grover_classifier/blob/main/README.md

S3.6 Chemprop

Model card – Chemprop

Model details

- **Model name:** Chemprop Message Passing Neural Network Tox21 Baseline
- **Developer:** MIT (trained by JKU Linz)
- **Paper URL:** https://pubs.acs.org/doi/full/10.1021/acs.jcim.3c01250
- **Model type / architecture:**
  - Message Passing Neural Network implemented using `Chemprop`.
  - **Hyperparameters:** https://huggingface.co/spaces/ml-jku/tox21_chemprop_classifier/blob/main/config/config.toml
  - A multitask network is trained for all Tox21 targets.
- **Inference:** Access via FastAPI endpoint. Upon receiving a Tox21 prediction request, the model generates and returns predictions for all Tox21 targets simultaneously.
- **Model version:** v0
- **Model date:** 14.10.2025
- **Reproducibility:** Code for full training is available and enables reproducible retraining of the model from scratch.

Intended use:
This model serves as a baseline benchmark for evaluating and comparing toxicity prediction methods across the 12 pathway assays of the Tox21 dataset. It is not intended for clinical decision-making without experimental validation.

Metric:
Each Tox21 task is evaluated using the area under the receiver operating characteristic curve (AUC). Overall performance is reported as the mean AUC across all individual tasks.

Training data:
Tox21 training and validation sets.
Evaluation data:
Tox21 test set.

Hugging Face Space:
https://huggingface.co/spaces/ml-jku/tox21_chemprop_classifier/blob/main/README.md

S3.7 Graph Isomorphism Network

Model card – GIN

Model details

- **Model name:** Graph Isomorphism Network Tox21 Baseline
- **Developer:** MIT & Stanford (trained by JKU Linz)
- **Paper URL:** https://arxiv.org/abs/1810.00826
- **Model type / architecture:**
  - Graph Isomorphism Network implemented using `PyTorch`.
  - Node features `data.x` are obtained using `torch_geometric.utils.from_rdmol`. Edge attributes were not used.

- **Hyperparameters:** https://huggingface.co/spaces/ml-jku/tox21_gin_classifier/blob/main/config/config.json
  - A multitask network is trained for all Tox21 targets.

- **Inference:** Access via FastAPI endpoint. Upon a Tox21 prediction request, the model generates and returns predictions for all Tox21 targets simultaneously.
- **Model version:** v0
- **Model date:** 14.10.2025
- **Reproducibility:** Code for full training is available and enables retraining from scratch.

Intended use:
This model serves as a baseline benchmark for evaluating and comparing toxicity prediction methods across the 12 pathway assays of the Tox21 dataset. It is not intended for clinical decision-making without experimental validation.

Metric:
Each Tox21 task is evaluated using the area under the receiver operating characteristic curve (AUC). Overall performance is reported as the mean AUC across all individual tasks.

Training data:
Tox21 training and validation sets.
Evaluation data:
Tox21 test set.

Hugging Face Space:
https://huggingface.co/spaces/ml-jku/tox21_gin_classifier/blob/main/README.md

S3.8 TabPFN

Model card – TabPFN

Model details

- **Model name:** TabPFN Tox21 Baseline
- **Developer:** PriorLabs (trained by JKU Linz)
- **Paper URL:** https://openreview.net/forum?id=eu9fVjVasr4
- **Model type / architecture:**
  - TabPFNv2 implemented using `tabpfn.TabPFNClassifier`.
  - **Hyperparameters**: https://huggingface.co/spaces/ml-jku/tox21_tabpfn_classifier/blob/main/config/config.json
  - A separate single-task TabPFNv2 model is trained for each Tox21 target.
- **Inference:** Local inference. Provide SMILES inputs to `predict()` in `predict.py` after completing setup and installation. A separate TabPFNv2 model is called for each target. Outputs are collected across all single-task models and returned.
- **Model version:** v0
- **Model date:** 12.10.2025
- **Reproducibility:** Code for full training is available and enables refitting of TabPFNv2 from scratch.
- **License / availability**: Open-weight model available under Prior Labs License

Intended use:
This model serves as a baseline benchmark for evaluating and comparing toxicity prediction methods across the 12 pathway assays of the Tox21 dataset. It is not intended for clinical decision-making without experimental validation.

Metric:
Each Tox21 task is evaluated using the area under the receiver operating characteristic curve (AUC). Overall performance is reported as the mean AUC across all individual tasks.

Training data:
Tox21 training and validation sets.
Evaluation data:
Tox21 test set.

Hugging Face Space:
https://huggingface.co/spaces/ml-jku/tox21_tabpfn_classifier/blob/main/README.md

S3.9 GPT-OSS

Model card – GPT-OSS

Model details

- **Model name:** GPT-OSS 120B
- **Developer:** OpenAI
- **Model card URL:** https://openai.com/index/gpt-oss-model-card
- **Model type / architecture:**
  - Mixture-of-Experts (MoE) Transformer architecture
  - 36 transformer layers, 117B total parameters (5.1B active per forward pass)
  - 128 experts per MoE layer with top-4 expert routing
  - Hidden dimension 2880, 64 attention heads, Grouped Query Attention (group size 8)
  - Context length 128K tokens
- **Inference:**
  - Context length 131,072 tokens
  - Temperature 0.7
  - Reasoning level: `high`
  - Prompting strategy: see Section S3.9
  - Inference engine: vLLM
  - Rollout strategy: five independent rollouts per molecule with aggregated predictions
- **Model version:** gpt-oss-120b
- **Model date:** August 5, 2025
- **License / availability:** Open-weight model available under the Apache 2.0 license; can be fine-tuned for specialized use cases.

Intended use:
This open-weight model is designed for reasoning, agentic, and developer tasks. It is intended for research and development purposes, including code generation, technical analysis, scientific reasoning, and complex problem solving.

Metric:
Each Tox21 task is evaluated using the area under the receiver-operating-characteristic curve (AUC). Overall performance is reported as the mean AUC across all tasks.

Training data:
Proprietary dataset (composition not publicly disclosed). Evaluated without additional fine-tuning or few-shot prompting.
Evaluation data:
Tox21 test set.

Hugging Face Model Repository

- **Repository:** https://huggingface.co/openai/gpt-oss-120b
- **Inference engine:** vLLM [6]

Prompt Template & Model Configuration

The language model was queried using a two-part prompt structure with system and user roles:

Prompt structure

System: You are an expert in molecular toxicity prediction. Analyze molecules and provide probability scores between `0.000` and `1.000`. Always respond with up to three decimal places.

User: Analyze whether this molecule is likely to be toxic in the `{target}` assay.

Target: `{description}`
SMILES: `{smiles}`

Provide only a probability between `0.000` and `1.000` indicating the likelihood of toxicity. Respond with only the number.

The template variables `{target}`, `{description}`, and `{smiles}` were populated for each prediction task. Table S1 lists all twelve Tox21 assay targets and their corresponding descriptions used in the prompts.

Model Configuration: We used the GPT-OSS 120B model [7] with default sampling settings as suggested by the model developers. This means a context size of 131,072 tokens, a sampling temperature of 0.7, and a reasoning setting of `high`. We perform five individual rollouts using vLLM [6] as our inference engine.

S3.10 Pretrained Encoders

This section provides a brief overview of the architectures of the pretrained encoders evaluated in this work.

- MolBERT [8] is a BERT-based transformer trained on molecular SMILES strings, consisting of 12 transformer layers with 12 attention heads, a hidden dimension of 768 and ∼85 million (M) parameters. Molecule representations were obtained from the pretrained encoder output.
- ChemBERTa-2 [9] is a RoBERTa-based molecular language model pretrained using masked lanuage modeling (MLM) and multi-task regression (MTR) on up to 77 million PubChem molecules using the SMILES representation. The model consists of 3 transformer layers with 12 attention heads, a hidden dimension of 384, and ∼3.4M parameters.
- ChemFM [10] is a large-scale causal decoder-only molecular foundation model based on the LLaMA architecture. The model comprises 30 transformer decoder layers, 48 attention heads, a hidden dimension of 3072, and 3 billion parameters.
- The Molecule Attention Transformer (MAT) [11] is a graph transformer that augments self-attention with interatomic distance information and molecular graph structure. The 20M parameter model employs 8 encoder layers with 16 attention heads, a hidden dimension of 1024, and 36-dimensional input atom features. Molecule-level representations are obtained by global mean pooling over the atom embeddings.
- The Relative Molecule Attention Transformer (R-MAT) [12] extends MAT by incorporating relative positional encodings and edge-aware attention mechanisms. The 4M-parameter model consists of 10 transformer encoder layers with 12 attention heads, a hidden dimension of 768, 37-dimensional input atom features, and 46-dimensional edge features. The encoder is followed by a 2-layer feed-forward network with a hidden dimension of 1536 and an output dimension of 768. Molecule-level embeddings are obtained using the GROVER-style graph aggregation module.

- Contrastive Language–Assay Molecule Pre-Training (CLAMP)[13] is a contrastive pretraining framework that aligns molecular and textual representations in a shared latent space. Its pretrained molecular encoder is a multilayer perceptron with ∼56M parameters, comprising 2 hidden layers of size 4096 and 2048, and producing a 768-dimensional molecular embedding. The encoder operates on an 8,912-dimensional molecular descriptor representation composed of Morgan and RDKit fingerprints.

S4 Additional results

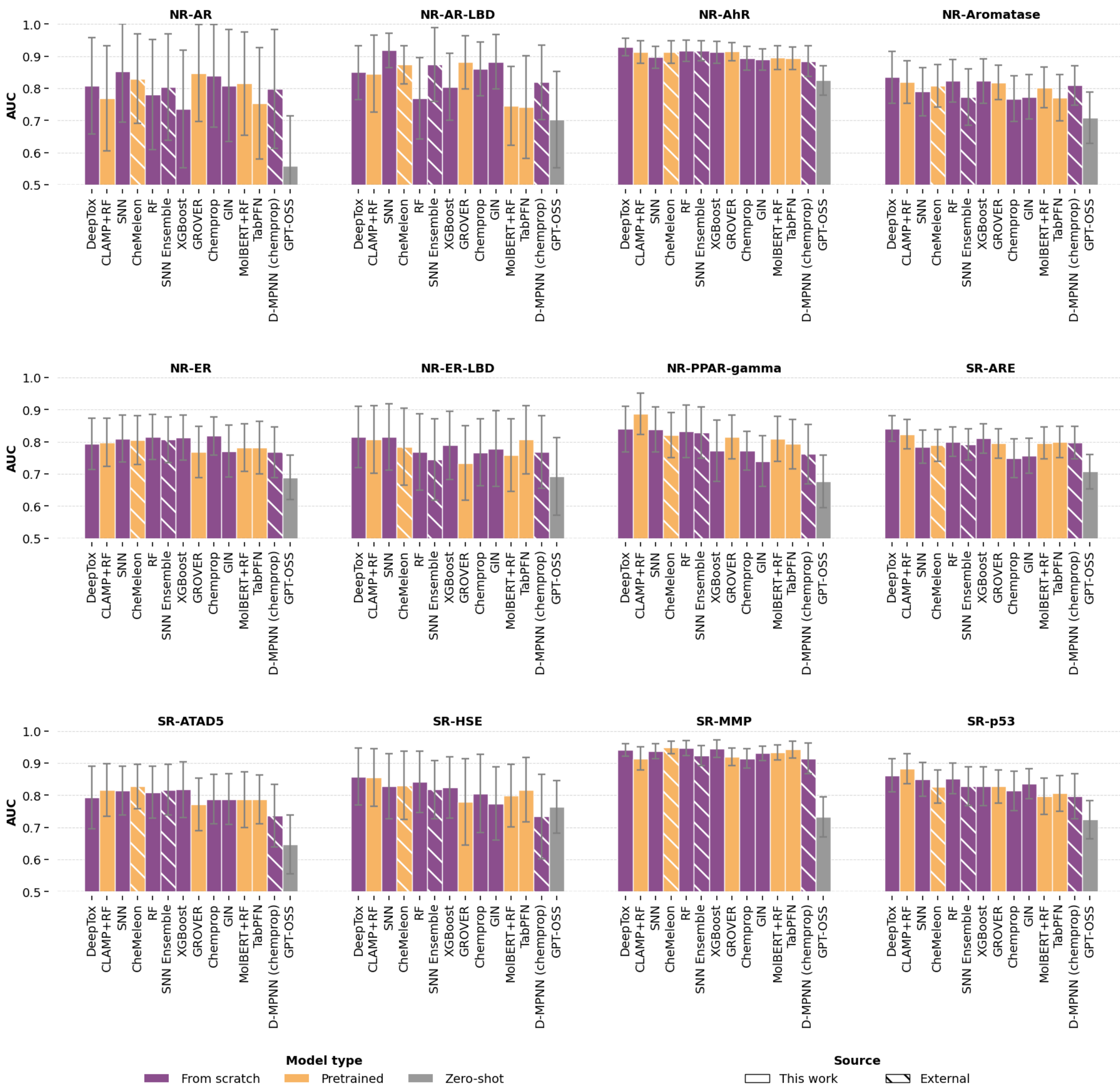

Figure S2: Task-wise comparison of model performance across the twelve Tox21 endpoints for multiple model types and developers. Reported values denote the test AUC of each submitted model with its 95% confidence interval, computed using DeLong's closed-form method.

Table S5: Performance of pretrained encoders on the Tox21 test set. Reported values denote the average ("overall") and task-wise test AUC across the twelve Tox21 endpoints, together with their 95% confidence intervals. Confidence intervals for the overall score were estimated using bootstrap resampling (5,000 iterations), whereas task-wise confidence intervals were computed using DeLong's closed-form method.

**Model**	**Overall**	**NR-AR**	**NR-AR-LBD**	**NR-AhR**	**NR-Aromatase**	**NR-ER**	**NR-ER-LBD**	**NR-PPAR-gamma**	**SR-ARE**	**SR-ATAD5**	**SR-HSE**	**SR-MMP**	**SR-p53**
MolBERT [1]	0.810<br>.784–.834	0.815<br>.654–.976	0.745<br>.622–.867	0.895<br>.858–.932	0.802<br>.738–.866	0.782<br>.707–.856	0.758<br>.645–.871	0.809<br>.739–.880	0.796<br>.746–.845	0.786<br>.699–.873	0.798<br>.701–.895	0.932<br>.909–.956	0.797<br>.740–.854
ChemBERTa [2]	0.784<br>.759–.808	0.725<br>.565–.885	0.744<br>.635–.853	0.896<br>.856–.936	0.773<br>.691–.855	0.773<br>.700–.846	0.678<br>.562–.795	0.779<br>.705–.853	0.763<br>.710–.817	0.798<br>.721–.874	0.788<br>.694–.883	0.929<br>.905–.953	0.762<br>.701–.823
ChemFM [3]	0.663<br>.632–.694	0.656<br>.472–.839	0.509<br>.311–.708	0.757<br>.703–.811	0.688<br>.604–.772	0.647<br>.566–.729	0.591<br>.443–.739	0.663<br>.580–.745	0.655<br>.599–.710	0.608<br>.515–.701	0.641<br>.521–.762	0.810<br>.761–.859	0.728<br>.666–.789
MAT [4]	0.778<br>.748–.809	0.716<br>.522–.910	0.579<br>.345–.814	0.892<br>.855–.930	0.799<br>.728–.870	0.748<br>.665–.832	0.754<br>.643–.866	0.731<br>.642–.821	0.766<br>.715–.817	0.785<br>.706–.864	0.788<br>.691–.885	0.938<br>.911–.966	0.842<br>.791–.894
R-MAT [5]	0.791<br>.765–.816	0.768<br>.611–.926	0.723<br>.587–.859	0.900<br>.861–.940	0.792<br>.720–.863	0.760<br>.679–.841	0.720<br>.603–.838	0.767<br>.679–.855	0.763<br>.711–.815	0.780<br>.697–.864	0.760<br>.653–.867	0.952<br>.933–.971	0.806<br>.748–.863
CLAMP [6]	0.844<br>.819–.867	0.769<br>.605–.933	0.845<br>.726–.965	0.913<br>.878–.947	0.819<br>.753–.886	0.798<br>.724–.872	0.807<br>.702–.912	0.886<br>.822–.950	0.823<br>.777–.869	0.816<br>.733–.899	0.855<br>.766–.944	0.914<br>.878–.950	0.883<br>.836–.930

[1] Fabian et al. [8], [2] ChemBERTa-77M-MTR [9], [3] ChemFM-3B [10], [4] MAT-20M [11], [5] R-MAT-4M [12], [6] Seidl et al. [13],

Table S6: Results of the Bayesian Bradley–Terry test (BBT) comparing the top-performing model with each of the remaining models on the leaderboard. For each comparison, the table reports the probability that the top-performing model is superior ($P_{\text{above 0.5}}$), the probability that the models are practically equivalent ($P_{\text{in ROPE}}$), and the corresponding weak interpretation.

**Pair**	$P_{\text{above 0.5}}$	$P_{\text{in ROPE}}$	**Weak Interpretation**
DeepTox > CLAMP	0.90	0.34	Inconclusive
DeepTox > CheMeleon	0.98	0.13	DeepTox better
DeepTox > SNN	0.99	0.09	DeepTox better
DeepTox > RF	1.00	0.05	DeepTox better
DeepTox > XGBoost	1.00	0.00	DeepTox better
DeepTox > SNN Ensemble	1.00	0.00	DeepTox better
DeepTox > GROVER	1.00	0.00	DeepTox better
DeepTox > TabPFN	1.00	0.00	DeepTox better
DeepTox > MolBERT	1.00	0.00	DeepTox better
DeepTox > Chemprop	1.00	0.00	DeepTox better
DeepTox > GIN	1.00	0.00	DeepTox better
DeepTox > D-MPNN (chemprop)	1.00	0.00	DeepTox better
DeepTox > GPT-OSS	1.00	0.00	DeepTox better

Table S7: Results of the Bayesian Bradley–Terry test (BBT) comparing the top five models on the current leaderboard with one another. For each comparison, the table reports the probability that the top-performing model is superior ($P_{\text{above 0.5}}$), the probability that the models are practically equivalent ($P_{\text{in ROPE}}$), and the corresponding weak interpretation.

**Pair**	$P_{\text{above 0.5}}$	$P_{\text{in ROPE}}$	**Weak Interpretation**
DeepTox > CLAMP	0.90	0.34	Inconclusive
DeepTox > CheMeleon	0.98	0.13	DeepTox better
DeepTox > SNN	0.99	0.09	DeepTox better
DeepTox > RF	1.0000	0.05	DeepTox better
CLAMP > CheMeleon	0.78	0.56	Inconclusive
CLAMP > SNN	0.83	0.48	Inconclusive
CLAMP > RF	0.91	0.34	Inconclusive
CheMeleon > SNN	0.58	0.68	Inconclusive
CheMeleon > RF	0.73	0.60	Inconclusive
SNN > RF	0.66	0.65	Inconclusive

S5 FastAPI Template Details

Overview. We provide a minimal FastAPI template that exposes a single batch inference endpoint, `/predict`. The endpoint accepts a list of SMILES strings and returns a nested dictionary of prediction scores, keyed first by input SMILES and then by assay target. The template is built in a modular way so that users can easily adapt it for their models.

FastAPI server. The template mounts `/predict` and delegates to a pure function `predict_fn`. Code is stored in `app.py` and does not need to be adapted by the users:

```
from fastapi import FastAPI
from pydantic import BaseModel
from typing import Dict, List

app = FastAPI()

class Request(BaseModel):
    smiles: List[str]

class Response(BaseModel):
    predictions: Dict[str, Dict[str, float]]
    model_info: Dict[str, str]

@app.post("/predict", response_model=Response)
def predict(request: Request):
    predictions = predict_fn(request.smiles)
    return {
        "predictions": predictions,
        "model_info": {"name": "tox21_model", "version": "1.0.0"},
    }
```

Toxicity prediction wrapper. The function `predict_fn` - stored in `predict.py` - encapsulates preprocessing and inference. Users should only replace the data preprocessing and model code, keeping the input/output contract unchanged:

```
# Pseudo code; for a working example see one of the baseline models
def predict_fn(smiles_list: list[str]) -> dict[str, dict[str, float]]:
    """Return {'<smiles>': {'<target>': <pred>}} for each input SMILES.
    """

    # --- Preprocessing (replace with your pipeline) ---
    scaler = load_pickle("assets/scaler.pkl")
    features = create_features(smiles_list, scaler=scaler)

    # --- Model (replace with your model) ---
    model = Model(seed=42)
    model.load_model("assets/trained_model")

    # --- Inference ---
    predictions = model(features)

    # --- Correct output format ---
    for smiles in smiles_list:
        for target in targets:
            output_dict[smiles][target] = predictions[...]

    return output_dict
```

Interface guarantees and validation. For leaderboard compatibility, the service must:

- Accept variable batch sizes (the orchestrator may split requests).
- Return predictions for *every* SMILES–target pair. Missing entries or NaN values cause the submission to be rejected.
- Be deterministic given fixed weights and preprocessing artifacts (set random seeds if necessary).

Example request:

```
curl -X POST https://ml-jku-tox21-gin-classifier.hf.space/predict \
     -H "Content-Type:  application/json" \
     -d '{"smiles":  ["CCO", "c1ccccc1"]}'
```

Returned JSON:


```
{
  "predictions": {"CCO":{"NR-AhR":0.005747087765485048,
                         "NR-AR":0.001738760736770928,
                         "NR-AR-LBD":0.00021425147133413702,
                         ...
                         "SR-p53":0.0007309493375942111},
                  "c1ccccc1":{
                         ...
                  }},,
  "model_info": {"name": "Tox21 GIN classifier", "version": "1.0.0"}
}
```


Supporting References

[1] Mitchell, M.; Wu, S.; Zaldivar, A.; Barnes, P.; Vasserman, L.; Hutchinson, B.; Spitzer, E.; Raji, I. D.; Gebru, T. Model cards for model reporting. Proceedings of the conference on fairness, accountability, and transparency. 2019; pp 220–229.

[2] Wu, Z.; Ramsundar, B.; Feinberg, E. N.; Gomes, J.; Geniesse, C.; Pappu, A. S.; Leswing, K.; Pande, V. MoleculeNet: a benchmark for molecular machine learning. *Chemical Science* **2018**, *9*, 513–530.

[3] Ramsundar, B.; Eastman, P.; Walters, P.; Pande, V.; Leswing, K.; Wu, Z. *Deep Learning for the Life Sciences*; O'Reilly Media, 2019; https://www.amazon.com/Deep-Learning-Life-Sciences-Microscopy/dp/1492039837.

[4] Riniker, S.; Landrum, G. A. Open-source platform to benchmark fingerprints for ligand-based virtual screening. *Journal of Cheminformatics* **2013**, *5*, 26.

[5] Orosz, A.; Héberger, K.; Rácz, A. Comparison of Descriptor- and Fingerprint Sets in Machine Learning Models for ADME-Tox Targets. *Frontiers in Chemistry* **2022**, *10*, Publisher: Frontiers.

[6] Kwon, W.; Li, Z.; Zhuang, S.; Sheng, Y.; Zheng, L.; Yu, C. H.; Gonzalez, J. E.; Zhang, H.; Stoica, I. Efficient Memory Management for Large Language Model Serving with PagedAttention. Proceedings of the ACM SIGOPS 29th Symposium on Operating Systems Principles. 2023.

[7] OpenAI gpt-oss-120b & gpt-oss-20b Model Card. 2025; https://arxiv.org/abs/2508.10925.

[8] Fabian, B.; Edlich, T.; Gaspar, H.; Segler, M.; Meyers, J.; Fiscato, M.; Ahmed, M. Molecular representation learning with language models and domain-relevant auxiliary tasks. *arXiv preprint arXiv:2011.13230* **2020**,

[9] Ahmad, W.; Simon, E.; Chithrananda, S.; Grand, G.; Ramsundar, B. Chemberta-2: Towards chemical foundation models. *arXiv preprint arXiv:2209.01712* **2022**,

[10] Cai, F.; Zacour, K.; Zhu, T.; Tzeng, T.-R.; Duan, Y.; Liu, L.; Pilla, S.; Li, G.; Luo, F. ChemFM as a scaling law guided foundation model pre-trained on informative chemicals. *Communications Chemistry* **2025**,

[11] Maziarka, Ł.; Danel, T.; Mucha, S.; Rataj, K.; Tabor, J.; Jastrzębski, S. Molecule attention transformer. *arXiv preprint arXiv:2002.08264* **2020**,

[12] Maziarka, Ł.; Majchrowski, D.; Danel, T.; Gaiński, P.; Tabor, J.; Podolak, I.; Morkisz, P.; Jastrzębski, S. Relative molecule self-attention transformer. *Journal of Cheminformatics* **2024**, *16*, 3.

[13] Seidl, P.; Vall, A.; Hochreiter, S.; Klambauer, G. Enhancing activity prediction models in drug discovery with the ability to understand human language. International Conference on Machine Learning. 2023; pp 30458–30490.
